# Supplementary material for: Heparin-based hydrogel scaffolding alters the transcriptomic profile and increases the chemoresistance of MDA-MB-231 triple-negative breast cancer cells
Source: Biomater Sci. 2020 Feb 13;8(10):2786–96. doi: 10.1039/c9bm01481k (PMC7497406; doi:10.1039/c9bm01481k)
Supplement: Supplementary file 2 [file BM-008-C9BM01481K-s002.zip › Supplementary File 4/HP-BhydrogelvControl/Pathways/my_analysis.Gsea.1545201079907/HALLMARK_INTERFERON_GAMMA_RESPONSE.html]

Details for gene set HALLMARK\_INTERFERON\_GAMMA\_RESPONSE[GSEA]

|  || Dataset | expr.class.cls#HYD\_versus\_CONTROL.class.cls#HYD\_versus\_CONTROL\_repos |
| Phenotype | class.cls#HYD\_versus\_CONTROL\_repos |
| Upregulated in class | CONTROL |
| GeneSet | HALLMARK\_INTERFERON\_GAMMA\_RESPONSE |
| Enrichment Score (ES) | -0.31802762 |
| Normalized Enrichment Score (NES) | -1.4840122 |
| Nominal p-value | 0.0 |
| FDR q-value | 0.020230923 |
| FWER p-Value | 0.131 |
Table: GSEA Results Summary

  

Fig 1: Enrichment plot: HALLMARK\_INTERFERON\_GAMMA\_RESPONSE      
 Profile of the Running ES Score & Positions of GeneSet Members on the Rank Ordered List

  

| PROBE | DESCRIPTION (from dataset) | GENE SYMBOL | GENE\_TITLE | RANK IN GENE LIST | RANK METRIC SCORE | RUNNING ES | CORE ENRICHMENT || 1 | PTPN6 | na |  |  | 544 | 2.288 | -0.0073 | No |
| 2 | RBCK1 | na |  |  | 817 | 2.014 | -0.0028 | No |
| 3 | RNF213 | na |  |  | 1270 | 1.704 | -0.0107 | No |
| 4 | UPP1 | na |  |  | 1307 | 1.685 | 0.0031 | No |
| 5 | RNF31 | na |  |  | 1752 | 1.469 | -0.0065 | No |
| 6 | NOD1 | na |  |  | 1791 | 1.446 | 0.0050 | No |
| 7 | PARP12 | na |  |  | 1935 | 1.394 | 0.0104 | No |
| 8 | NLRC5 | na |  |  | 2222 | 1.265 | 0.0072 | No |
| 9 | OGFR | na |  |  | 2536 | 1.162 | 0.0016 | No |
| 10 | TRIM26 | na |  |  | 2685 | 1.122 | 0.0043 | No |
| 11 | IRF7 | na |  |  | 3003 | 1.036 | -0.0027 | No |
| 12 | NFKB1 | na |  |  | 3668 | 0.878 | -0.0295 | No |
| 13 | IL18BP | na |  |  | 3702 | 0.870 | -0.0231 | No |
| 14 | LATS2 | na |  |  | 4016 | 0.809 | -0.0320 | No |
| 15 | MARCH1 | na |  |  | 4136 | 0.782 | -0.0310 | No |
| 16 | TRIM14 | na |  |  | 4153 | 0.778 | -0.0245 | No |
| 17 | STAT2 | na |  |  | 4243 | 0.764 | -0.0221 | No |
| 18 | ZNFX1 | na |  |  | 4372 | 0.737 | -0.0220 | No |
| 19 | CIITA | na |  |  | 4409 | 0.732 | -0.0170 | No |
| 20 | VAMP5 | na |  |  | 4604 | 0.692 | -0.0208 | No |
| 21 | SOCS3 | na |  |  | 4653 | 0.684 | -0.0169 | No |
| 22 | PELI1 | na |  |  | 4774 | 0.664 | -0.0171 | No |
| 23 | ISG15 | na |  |  | 4916 | 0.639 | -0.0185 | No |
| 24 | IFI30 | na |  |  | 5258 | 0.592 | -0.0309 | No |
| 25 | MX1 | na |  |  | 5402 | 0.570 | -0.0331 | No |
| 26 | IRF1 | na |  |  | 5648 | 0.533 | -0.0410 | No |
| 27 | PML | na |  |  | 5893 | 0.500 | -0.0492 | No |
| 28 | OASL | na |  |  | 6099 | 0.470 | -0.0556 | No |
| 29 | SLC25A28 | na |  |  | 6103 | 0.470 | -0.0514 | No |
| 30 | GCH1 | na |  |  | 6157 | 0.463 | -0.0498 | No |
| 31 | ST3GAL5 | na |  |  | 6208 | 0.458 | -0.0482 | No |
| 32 | PTGS2 | na |  |  | 6325 | 0.440 | -0.0502 | No |
| 33 | HERC6 | na |  |  | 6366 | 0.434 | -0.0482 | No |
| 34 | TRIM25 | na |  |  | 6494 | 0.416 | -0.0510 | No |
| 35 | SP110 | na |  |  | 6691 | 0.394 | -0.0577 | No |
| 36 | BANK1 | na |  |  | 6842 | 0.379 | -0.0620 | No |
| 37 | CXCL10 | na |  |  | 6890 | 0.372 | -0.0610 | No |
| 38 | TNFAIP3 | na |  |  | 6980 | 0.364 | -0.0623 | No |
| 39 | SSPN | na |  |  | 7075 | 0.351 | -0.0640 | No |
| 40 | FGL2 | na |  |  | 7089 | 0.349 | -0.0614 | No |
| 41 | PTPN2 | na |  |  | 7106 | 0.347 | -0.0590 | No |
| 42 | LYSMD2 | na |  |  | 7230 | 0.334 | -0.0624 | No |
| 43 | IL6 | na |  |  | 7244 | 0.333 | -0.0600 | No |
| 44 | DHX58 | na |  |  | 7492 | 0.309 | -0.0701 | No |
| 45 | JAK2 | na |  |  | 7561 | 0.298 | -0.0708 | No |
| 46 | HLA-G | na |  |  | 7771 | 0.277 | -0.0793 | No |
| 47 | MVP | na |  |  | 7798 | 0.273 | -0.0781 | No |
| 48 | PIM1 | na |  |  | 8219 | 0.229 | -0.0980 | No |
| 49 | HLA-DQA1 | na |  |  | 8229 | 0.229 | -0.0964 | No |
| 50 | IL7 | na |  |  | 8303 | 0.219 | -0.0981 | No |
| 51 | PSMB10 | na |  |  | 8309 | 0.219 | -0.0964 | No |
| 52 | C1S | na |  |  | 8670 | 0.184 | -0.1136 | No |
| 53 | ADAR | na |  |  | 8794 | 0.167 | -0.1185 | No |
| 54 | SECTM1 | na |  |  | 8859 | 0.158 | -0.1204 | No |
| 55 | IL4R | na |  |  | 9138 | 0.135 | -0.1337 | No |
| 56 | SOCS1 | na |  |  | 9324 | 0.113 | -0.1424 | No |
| 57 | CMKLR1 | na |  |  | 9419 | 0.102 | -0.1464 | No |
| 58 | IL15RA | na |  |  | 9909 | 0.053 | -0.1716 | No |
| 59 | ICAM1 | na |  |  | 9949 | 0.046 | -0.1732 | No |
| 60 | OAS3 | na |  |  | 10110 | 0.027 | -0.1814 | No |
| 61 | CFB | na |  |  | 10199 | 0.015 | -0.1858 | No |
| 62 | CMPK2 | na |  |  | 10261 | 0.015 | -0.1889 | No |
| 63 | IFI44L | na |  |  | 10300 | 0.015 | -0.1908 | No |
| 64 | CASP1 | na |  |  | 10302 | 0.015 | -0.1907 | No |
| 65 | IFI35 | na |  |  | 10456 | 0.000 | -0.1987 | No |
| 66 | PTPN1 | na |  |  | 10546 | -0.009 | -0.2033 | No |
| 67 | MX2 | na |  |  | 10637 | -0.021 | -0.2078 | No |
| 68 | TAP1 | na |  |  | 10646 | -0.022 | -0.2081 | No |
| 69 | PARP14 | na |  |  | 10807 | -0.039 | -0.2161 | No |
| 70 | CASP8 | na |  |  | 10884 | -0.051 | -0.2196 | No |
| 71 | LY6E | na |  |  | 11120 | -0.081 | -0.2312 | No |
| 72 | APOL6 | na |  |  | 11145 | -0.085 | -0.2317 | No |
| 73 | CD274 | na |  |  | 11286 | -0.087 | -0.2382 | No |
| 74 | HLA-DMA | na |  |  | 11401 | -0.101 | -0.2433 | No |
| 75 | PFKP | na |  |  | 11433 | -0.105 | -0.2439 | No |
| 76 | HLA-B | na |  |  | 11495 | -0.110 | -0.2461 | No |
| 77 | NUP93 | na |  |  | 11519 | -0.114 | -0.2462 | No |
| 78 | TAPBP | na |  |  | 11603 | -0.126 | -0.2494 | No |
| 79 | CDKN1A | na |  |  | 11746 | -0.145 | -0.2555 | No |
| 80 | ITGB7 | na |  |  | 12009 | -0.173 | -0.2677 | No |
| 81 | C1R | na |  |  | 12054 | -0.180 | -0.2683 | No |
| 82 | MYD88 | na |  |  | 12113 | -0.189 | -0.2696 | No |
| 83 | IRF2 | na |  |  | 12188 | -0.198 | -0.2717 | No |
| 84 | FPR1 | na |  |  | 12252 | -0.207 | -0.2730 | No |
| 85 | LGALS3BP | na |  |  | 12273 | -0.209 | -0.2721 | No |
| 86 | NFKBIA | na |  |  | 12331 | -0.218 | -0.2731 | No |
| 87 | CCL2 | na |  |  | 12375 | -0.222 | -0.2733 | No |
| 88 | IFNAR2 | na |  |  | 12530 | -0.229 | -0.2793 | No |
| 89 | FAS | na |  |  | 12828 | -0.261 | -0.2924 | No |
| 90 | HLA-DRB1 | na |  |  | 12833 | -0.262 | -0.2902 | No |
| 91 | XAF1 | na |  |  | 12876 | -0.268 | -0.2899 | No |
| 92 | GBP4 | na |  |  | 12911 | -0.273 | -0.2892 | No |
| 93 | TRIM21 | na |  |  | 12921 | -0.274 | -0.2871 | No |
| 94 | CFH | na |  |  | 12948 | -0.278 | -0.2859 | No |
| 95 | CD74 | na |  |  | 12971 | -0.280 | -0.2844 | No |
| 96 | CXCL11 | na |  |  | 13037 | -0.289 | -0.2851 | No |
| 97 | IFIH1 | na |  |  | 13280 | -0.322 | -0.2949 | No |
| 98 | CD40 | na |  |  | 13309 | -0.327 | -0.2933 | No |
| 99 | SPPL2A | na |  |  | 13507 | -0.354 | -0.3003 | No |
| 100 | RAPGEF6 | na |  |  | 13529 | -0.357 | -0.2981 | No |
| 101 | IRF9 | na |  |  | 13588 | -0.367 | -0.2977 | No |
| 102 | SOD2 | na |  |  | 13651 | -0.378 | -0.2975 | No |
| 103 | ISG20 | na |  |  | 13723 | -0.391 | -0.2976 | No |
| 104 | PLA2G4A | na |  |  | 13844 | -0.411 | -0.3000 | No |
| 105 | PNPT1 | na |  |  | 13873 | -0.414 | -0.2977 | No |
| 106 | NCOA3 | na |  |  | 14080 | -0.428 | -0.3045 | No |
| 107 | NMI | na |  |  | 14136 | -0.432 | -0.3034 | No |
| 108 | DDX60 | na |  |  | 14208 | -0.443 | -0.3030 | No |
| 109 | PSMA2 | na |  |  | 14390 | -0.475 | -0.3080 | No |
| 110 | TRAFD1 | na |  |  | 14581 | -0.503 | -0.3133 | Yes |
| 111 | ARID5B | na |  |  | 14668 | -0.519 | -0.3130 | Yes |
| 112 | RIPK1 | na |  |  | 14682 | -0.520 | -0.3089 | Yes |
| 113 | RSAD2 | na |  |  | 14852 | -0.544 | -0.3127 | Yes |
| 114 | RTP4 | na |  |  | 14854 | -0.544 | -0.3077 | Yes |
| 115 | PSMA3 | na |  |  | 15032 | -0.567 | -0.3117 | Yes |
| 116 | STAT3 | na |  |  | 15037 | -0.567 | -0.3066 | Yes |
| 117 | IL15 | na |  |  | 15098 | -0.579 | -0.3044 | Yes |
| 118 | PSME2 | na |  |  | 15141 | -0.587 | -0.3011 | Yes |
| 119 | BST2 | na |  |  | 15210 | -0.597 | -0.2991 | Yes |
| 120 | PSMB8 | na |  |  | 15412 | -0.636 | -0.3038 | Yes |
| 121 | PDE4B | na |  |  | 15522 | -0.656 | -0.3034 | Yes |
| 122 | UBE2L6 | na |  |  | 15591 | -0.666 | -0.3007 | Yes |
| 123 | LAP3 | na |  |  | 15631 | -0.674 | -0.2965 | Yes |
| 124 | PSMB9 | na |  |  | 15703 | -0.682 | -0.2939 | Yes |
| 125 | BTG1 | na |  |  | 15833 | -0.711 | -0.2940 | Yes |
| 126 | ISOC1 | na |  |  | 15866 | -0.717 | -0.2890 | Yes |
| 127 | IFITM3 | na |  |  | 15996 | -0.744 | -0.2889 | Yes |
| 128 | EIF2AK2 | na |  |  | 16137 | -0.775 | -0.2890 | Yes |
| 129 | PNP | na |  |  | 16242 | -0.798 | -0.2871 | Yes |
| 130 | CASP4 | na |  |  | 16359 | -0.824 | -0.2855 | Yes |
| 131 | EIF4E3 | na |  |  | 16382 | -0.828 | -0.2789 | Yes |
| 132 | ST8SIA4 | na |  |  | 16410 | -0.834 | -0.2726 | Yes |
| 133 | WARS | na |  |  | 16504 | -0.855 | -0.2695 | Yes |
| 134 | TNFSF10 | na |  |  | 16587 | -0.869 | -0.2657 | Yes |
| 135 | RIPK2 | na |  |  | 16616 | -0.878 | -0.2590 | Yes |
| 136 | IFITM2 | na |  |  | 16644 | -0.884 | -0.2522 | Yes |
| 137 | SLAMF7 | na |  |  | 16677 | -0.892 | -0.2455 | Yes |
| 138 | SAMHD1 | na |  |  | 16685 | -0.894 | -0.2376 | Yes |
| 139 | STAT1 | na |  |  | 16812 | -0.929 | -0.2355 | Yes |
| 140 | SAMD9L | na |  |  | 16828 | -0.934 | -0.2276 | Yes |
| 141 | IFI44 | na |  |  | 16919 | -0.962 | -0.2234 | Yes |
| 142 | TDRD7 | na |  |  | 16953 | -0.970 | -0.2161 | Yes |
| 143 | OAS2 | na |  |  | 16982 | -0.981 | -0.2084 | Yes |
| 144 | MTHFD2 | na |  |  | 16986 | -0.982 | -0.1994 | Yes |
| 145 | VCAM1 | na |  |  | 16995 | -0.984 | -0.1907 | Yes |
| 146 | ARL4A | na |  |  | 17123 | -1.023 | -0.1878 | Yes |
| 147 | MT2A | na |  |  | 17171 | -1.038 | -0.1806 | Yes |
| 148 | IFIT1 | na |  |  | 17218 | -1.058 | -0.1732 | Yes |
| 149 | BATF2 | na |  |  | 17399 | -1.118 | -0.1722 | Yes |
| 150 | PLSCR1 | na |  |  | 17416 | -1.120 | -0.1626 | Yes |
| 151 | PSMB2 | na |  |  | 17433 | -1.128 | -0.1530 | Yes |
| 152 | TXNIP | na |  |  | 17600 | -1.197 | -0.1506 | Yes |
| 153 | PSME1 | na |  |  | 17774 | -1.276 | -0.1478 | Yes |
| 154 | IFI27 | na |  |  | 17788 | -1.281 | -0.1365 | Yes |
| 155 | USP18 | na |  |  | 17930 | -1.341 | -0.1314 | Yes |
| 156 | SRI | na |  |  | 17999 | -1.383 | -0.1221 | Yes |
| 157 | DDX58 | na |  |  | 18014 | -1.392 | -0.1099 | Yes |
| 158 | IFIT2 | na |  |  | 18078 | -1.429 | -0.0999 | Yes |
| 159 | TOR1B | na |  |  | 18209 | -1.505 | -0.0927 | Yes |
| 160 | CASP7 | na |  |  | 18210 | -1.506 | -0.0786 | Yes |
| 161 | NAMPT | na |  |  | 18342 | -1.596 | -0.0707 | Yes |
| 162 | HIF1A | na |  |  | 18348 | -1.603 | -0.0560 | Yes |
| 163 | IFIT3 | na |  |  | 18445 | -1.669 | -0.0455 | Yes |
| 164 | BPGM | na |  |  | 18542 | -1.737 | -0.0343 | Yes |
| 165 | CASP3 | na |  |  | 18683 | -1.879 | -0.0242 | Yes |
| 166 | B2M | na |  |  | 19069 | -2.604 | -0.0202 | Yes |
| 167 | VAMP8 | na |  |  | 19121 | -2.887 | 0.0040 | Yes |
Table: GSEA details [plain text format]

  

Fig 2: HALLMARK\_INTERFERON\_GAMMA\_RESPONSE      
 Blue-Pink O' Gram in the Space of the Analyzed GeneSet

  

Fig 3: HALLMARK\_INTERFERON\_GAMMA\_RESPONSE: Random ES distribution      
 Gene set null distribution of ES for **HALLMARK\_INTERFERON\_GAMMA\_RESPONSE**
